# Adipogenesis biomarkers as the independent predictive factors for breast cancer recurrence: a systematic review and meta-analysis

**DOI:** 10.1186/s12885-024-12931-1

**Published:** 2024-09-27

**Authors:** Shihang Hu, Sze Keong Tey, Ava Kwong

**Affiliations:** Department of Surgery, Li Ka Shing Faculty of Medicine, The University of Hong Kong, Hong Kong SAR, China

**Keywords:** Breast cancer, Recurrence, Adipogenesis, Prediction

## Abstract

**Background:**

Comprehensive analysis of clinical evidence for breast cancer adipogenesis with prognosis is lacking. This study aims to consolidate the latest evidence on the relationship between adipogenesis and breast cancer outcomes.

**Data sources:**

: Medline, Web of Science, Embase, Scopus, Clinicaltrials.gov, Cochrane library.

**Methods:**

A systematic review was conducted according to the PRISMA guidelines. Studies that reported the correlation between tumor adipogenesis and cancer recurrence or empirical pathological markers were included for meta-analysis. The standard reference for pathological markers determination was set as histopathological examination. The PROSPERO ID was CRD489135.

**Results:**

Eleven studies were included in this systematic review and meta-analysis. Several adipogenesis biomarkers involved in the synthesis, elongation, and catabolism of fatty acids, such as FASN, Spot 14, pS6K1, lipin-1, PLIN2, Elovl6, and PPARγ, were identified as the potential biomarkers for predicting outcomes. Through meta-analysis, the predictive value of adipogenesis biomarkers for 5-year recurrence rate was calculated, with a pooled predictive risk ratio of 2.19 (95% CI: 1.11–4.34). In terms of empirical pathological markers, a negative correlation between adipogenesis biomarkers and ki-67 was observed (RR: 0.69, 95% CI: 0.61–0.79). However, no significant correlation was found between the adipogenesis and ER, PR, HER2, or p53 positivity.

**Conclusions:**

Biomarker of adipogenesis in breast cancer is a significant predictor of long-term recurrence, and this prediction is independent of HR, HER2, and ki-67. The diverse roles of adipogenesis in different breast cancer subtypes highlight the need for further research to uncover specific biomarkers that can used for diagnosis and prediction.

**Protocol registration:**

*PROSPERO ID: CRD489135*.

**Supplementary Information:**

The online version contains supplementary material available at 10.1186/s12885-024-12931-1.

## Introduction

Breast cancer continue to pose a significant threat to women’s lives and health, and its prognosis is influenced by a multitude of genetic and non-genetic factors [1]. While clinicopathological factors such as TNM stage and grade have traditionally been considered, established molecular biomarkers now play a crucial role in determining prognosis and predicting treatment response. Molecular markers like estrogen receptor (ER), progesterone receptor (PR), human epidermal growth factor receptor 2 (HER2), Ki67 proliferation marker, and more recently BRCA1/2 gene, cyclin D1, VEGF, and TOPOII have been extensively studied and established for their prognostic value [2–8]. However, these conventional biomarkers are not sufficient to precisely guide treatment decisions and predict prognosis. They only provide insights into the tumor’s biological behavior at a specific moment in time [9]. Considering the high heterogeneity of breast cancer and the significance of prognostic markers in patient management, it becomes crucial to enhance the prognosis evaluation system. This improvement would enable more accurate prediction of treatment response and facilitate the selection of optimized treatment strategies.

In recent years, there has been a growing interest in the role of adipogenesis in breast cancer due to the unique microenvironment of breast cancer that is closely associated with surrounding adipose tissue. The study of the mechanism of invasion and metastasis of breast cancer has increased its importance particularly due to more clinical applications [10]. Studies have confirmed that high adipogenesis activity is linked to cancer progression, recurrence, and metastasis [11–14]. Previous research has demonstrated that most breast cancer cells exhibit an “adipogenic” phenotype, which is characterized by increased lipogenesis and a dependency on fatty acid synthesis for growth and survival [15]. Compared to other cancers, breast cancer is surrounded by numerous fat pads, providing a basic niche for tumor initiation and progression [15]. Additionally, adipogenesis has been identified as a metabolic pathway of drug resistance in breast cancer chemotherapy, endocrine therapy, and HER2 targeted therapy. Thus, adipogenesis may be a therapeutic barrier as it is involved in the resistance mechanism to various therapies for breast cancer [16–18]. Despite these findings, the molecular link between adipogenesis and breast cancer is not yet fully understood.

Recent studies have demonstrated that different breast cancer subtypes exhibit specific adipogenic phenotypes that can meet their unique metabolic needs. For instance, luminal subtypes rely on de novo adipogenesis (DNL) to meet their biomass and energy demands, while basal-like subtype utilize exogenous fatty acids and triacylglycerol synthesis [19]. In HER2 positive breast cancer, adipogenesis plays a more significant role than other subtypes due to the upregulation of fatty acid synthase(FASN) transcription by the HER2 gene, leading to an increase in de novo fatty acid synthesis [20]. Conversely, adipogenesis in triple-negative breast cancer (TNBC) is typically reduced, although high adipogenesis TNBC enriches the gene set related to fat metabolism, rather than cell proliferation or inflammation gene sets [21]. Given the critical role of adipogenesis in breast cancer, the key signaling pathways involved in this process could serve as new biomarkers for predicting oncological outcomes and guiding therapeutic decision-making, and the manipulation of lipid metabolism holds potential as a new therapeutic approach for anti-cancer treatment.

Over the past few decades, significant efforts have been made to explore and incorporate the use of breast cancer biomarkers in order to improve prognostic evaluation. While several studies have investigated biomarkers related to adipogenesis in breast cancer lipid metabolism, there is a lack of large-scale clinical studies conducted across multiple centers. Furthermore, no breast cancer- specific adipogenesis biomarkers have been included in clinical guidelines for prognostic evaluation and treatment decision-making. Consequently, the objective of this study is to conduct an evidence-based investigation into the clinical relevance of adipogenesis in breast cancer prognosis through a systematic literature review and meta-analysis. The findings of this study aim to provide valuable evidence for the future development of clinical guidelines in this field.

## Methods

### Protocol and registration

The protocol for this systematic review has been registered in the International Prospective register of Systematic Reviews, with the PROSPERO ID CRD489135. The systematic review adhered to the guidelines outlined in the Preferred Reporting Items for Systematic Reviews and Meta-Analyses (PRISMA).

### Eligibility criteria

All studies that met the following criteria were included for in-depth review, data extraction, and analysis:

a) Clinical study reporting on the correlation between the histological biomarker of intratumor adipogenesis and breast cancer outcomes.

b) Adipogenesis was defined using immunohistochemistry staining for a specific biomarker.

c) Measured outcomes included cancer recurrence, overall survival, or positivity of histological markers (ER, PR, HER-2, Ki-67, etc.).

d) Data to generate a complete contingency table for each outcome was provided.

e) The study was published in the English language.

Literature sources and Search strategy.

The literature search was conducted in Medline, Embase, Web of Science, SCOPUS, Clinicaltrials.gov, and Cochrane library. The search was first conducted in July 2023 and was updated in April of 2024. Search strategy was set as: ((breast cancer) OR (breast tumor) OR (mammary tumor) OR (mammary cancer)) AND ((adipogenesis) OR (lipogenesis) OR (adipogenic) OR (adipogenic differentiation)).

### Study selection and methodology quality assessment

The literature identified through the search strategy was initially screened to remove duplicates across databases and studies that were not focused on breast cancer. Review articles, case reports, and studies conducted only on animal or in vitro without patient inclusion were also excluded. The abstracts and full texts of the remaining publications were then reviewed to exclude studies that were unrelated to cancer adipogenesis, did not report any outcomes, or did not provide sufficient data for meta-analysis. Information extracted from the included publications included the author, year of publication, region of recruited patients, study design, number of patients, breast cancer type or subtype, measured outcomes, and biomarkers with their predefined cut-offs. The methodology of the included studies was assessed for quality using the QUADAS-2 criteria.

### Statistical methods

The raw data for true positive (TP), false positive (FP), true negative (TN), and false negative (FN) of the biomarkers were extracted from the published data of the included studies. The diagnostic or predictive value of the biomarkers was evaluated by calculating the sensitivity, specificity, positive predictive value (PPV), negative predictive value (NPV), and likelihood ratios (+ LR, -LR). To assess the diagnostic accuracy and heterogeneity of the included studies during meta-analysis, a summary receiver operating characteristic (SROC) curve, and funnel plot were employed. All statistical analyses and figures were generated using Review Manager (RevMan 5.4.1, The Cochrane Collaboration).

## Results

### Study selection

**Fig. 1:**
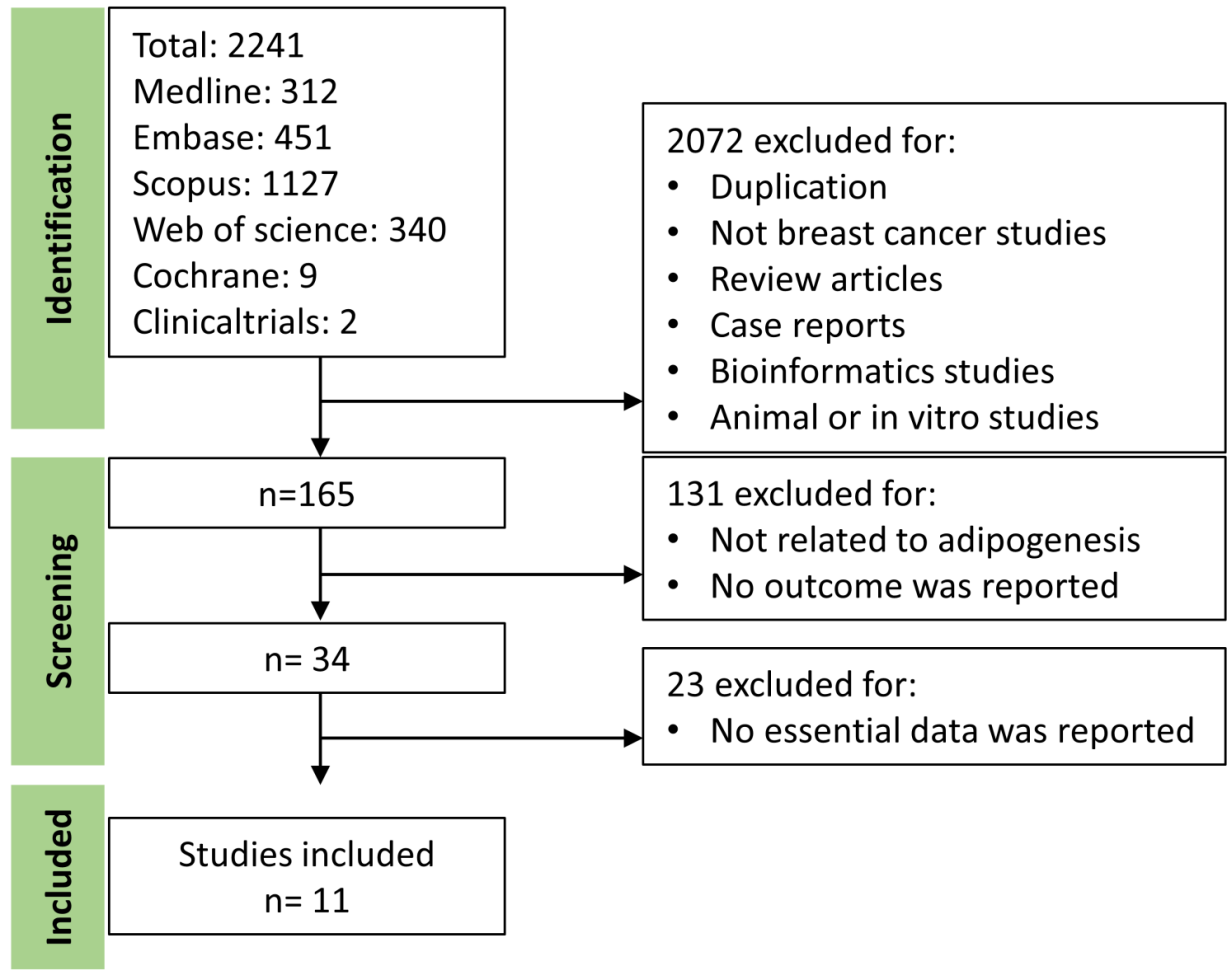
Selection flow for publications

Out of 2,265 articles that were screened initially, 2092 publications were excluded based on their titles and abstracts due to duplication, not being focused on breast cancer subjects, being animal or in vitro studies, or being review articles or case reports, among other reasons. An additional 162 publications were excluded because they were not related to tumor adipogenesis, did not report any clinical outcomes, or did not provide essential data for analysis. Ultimately, 11 cohort studies were included for the systematic review and meta-analysis. Additional 3 bioinformatics studies using public database were also included for validation. The selection workflow and results are presented in Fig. 1.

### Risk of bias

**Fig. 2:**
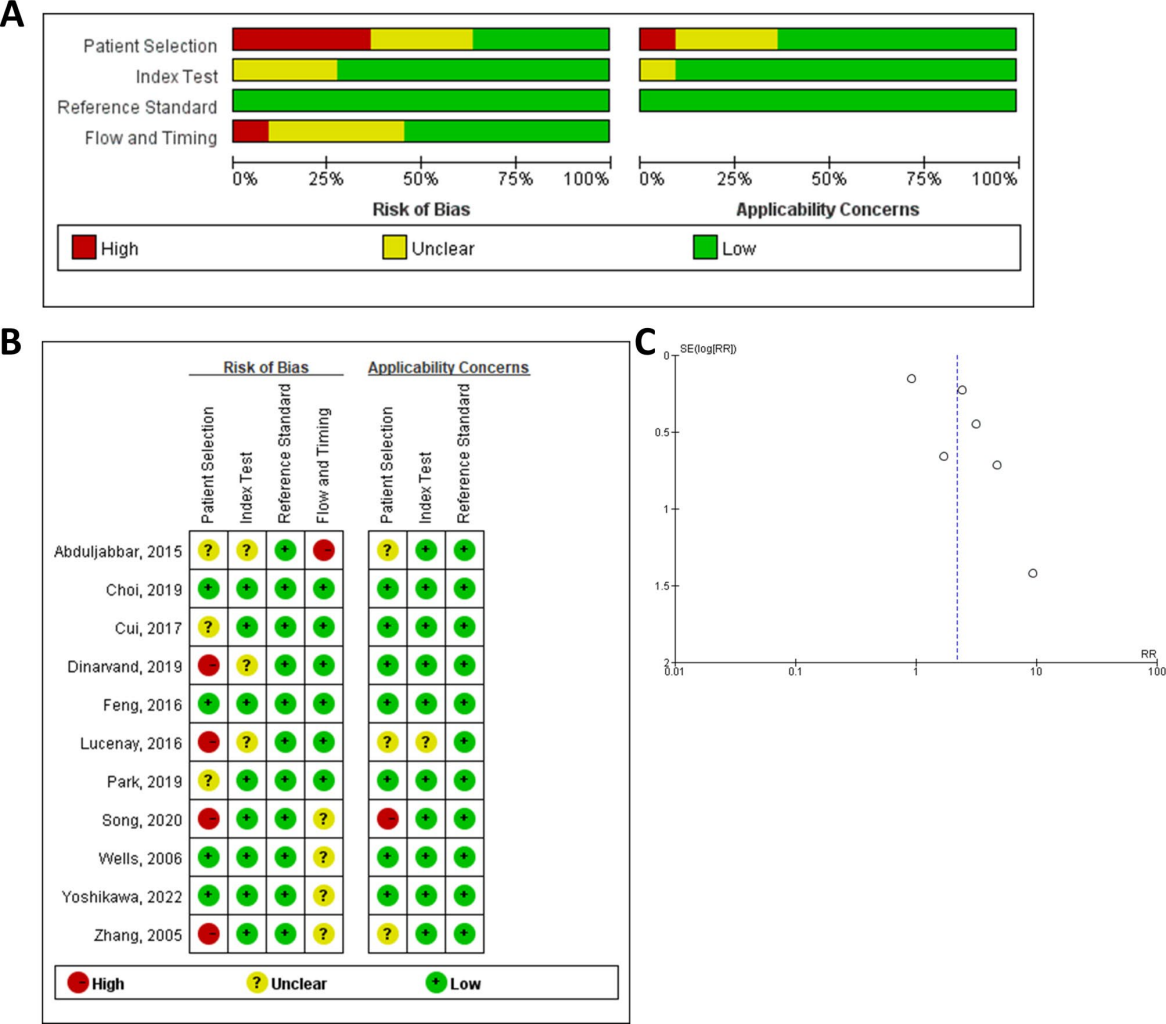
Quality evaluation and publication bias for included studies. (**A**) Overview of the methodology bias; (**B**) methodology bias for individual publication; (**C**) funnel plots for publication bias

The risk of bias in all studies was assessed using the Quality Assessment of Diagnostic Accuracy Studies – 2 (QUADAS-2) tool by two independent researchers. The summary of the pooled results is presented in Fig. 2, which indicates that the methodology bias was low. Publication bias was analyzed by funnel plot, Egger’s and Begg’s tests (Fig. 2, Supplementary Table 1), which showed low bias.

### Study characteristics

A total of 11 studies were included in the systematic review [12, 14, 20, 22–29]. Table 1 and 2 provides a summary of the included studies, including first author and country, year of publication, study design, biomarker studied, number of patients, and pathology subtypes, as well as the evaluated endpoints. Among the included studies, 10 were retrospective studies, while only 1 was a prospective cohort study. The biomarkers investigated for tumor adipogenesis included (a) fatty acid synthase (FASN), (b) Spot 14 (S14), (c) phosphorylated ribosomal protein S6 kinase-1 (pS6K1), (d) lipin-1, (e) adipophilin (PLIN2), (f) Elongation of long chain fatty acids family member 6 (Elovl6), and (g) peroxisome proliferator-activated receptor-gamma (PPARγ). Immunohistochemistry staining on tumor tissue was used to examine all these biomarkers, with a specific predefined cut-off for staining score. However, Dinarvand et al. reported the predictive value of lipin-1 using a messenger RNA (mRNA) cut-off [23].

**Table 1:** Characteristics of included studies

No.	Year	Author	Country	Study design	Biomarker	Total patients, *n*	Adipogenesis-high patients, *n*	Setting	Endpoints
1	2005	Zhang, et al.[21]	Singapore	Retrospective cohort	Tumor tissue FASN protein	87	63	HER-2+/- BC	-
2	2006	Wells, et al.[19]	USA	Retrospective cohort	Tumor tissue Spot 14 (THRSP)	88	67	DCIS; Node- BC; Node + invasive BC	5-year RFS
3	2015	Abduljabbar, et al. [12]	UK	Retrospective cohort	Tumor tissue PPARγ	1100	320	Luminal ER + BC, hormone therapy	15-year recurrence
4	2016	Lucenay, et al. [16]	USA	Prospective cohort	Tumor tissue PLIN2 (adipophilin)	100	29	Stage I-III BC	2 to 10-year RFS
5	2016	Feng, et al. [15]	Taiwan, China	Retrospective cohort	Tumor tissue Elovl6	70	26	BC patients post mastectomy; All BC/ER+/PR+	5-year RFS
6	2017	Cui, et al. [13]	China	Retrospective cohort	Tumor tissue FASN	50	35	Not defined	Overall recurrence
7	2019	Choi, et al. [10]	Korea	Retrospective cohort	Tumor tissue pS6K1	428	244	ER + Node + BC, hormone therapy	5-year RFS
8	2019	Park, et al. [17]	Korea	Retrospective cohort	Tumor tissue pS6K1	296	219	HR + HER2- BC, hormone therapy	5/10-year RFS
9	2019	Dinarvand, et al. [14]	Iran	Retrospective cohort	Tumor tissue Lipin-1 mRNA	55	26	All BCs	-
10	2020	Song, et al. [18]	China	Retrospective cohort	Tumor tissue lipin-1 protein	60	29	All BCs	5-year OS; 5-year RFS
11	2022	Yoshikawa, et al. [20]	Japan	Retrospective cohort	Tumor tissue FASN	61	35	TNBC	5/10-year RFS

*Abbreviations* FASN, fatty acid synthase; S14, Spot 14; pS6K1, phosphorylated ribosomal protein S6 kinase-1; PLIN2, adipophilin; Elovl6, elongation of long chain fatty acids family member 6; PPARγ, peroxisome proliferator-activated receptor-gamma; ER, estrogen receptor; PR, progesterone receptor; HER2, human epidermal growth factor receptor 2; BC, breast cancer; OS, overall survival; RFS, recurrence-free survival; TNBC, triple-negative breast cancer; DCIS, ductal carcinoma in situ

**Table 2:** Statistics and diagnostic accuracy of included studies

No.	Author, date	Biomarker	Cut-off	Outcome	TP	FP	FN	TN	Sensitivity (95% CI), %	Specificity (95% CI), %	PPV	NPV	+LR	-LR
1	Zhang [21], 2005	FASN	IHC score 2–3 vs. 0–1	HER-2 positivity	17	46	4	20	80.95 (58.09–94.55)	30.30 (19.59–42.85)	0.27	0.83	1.16	0.63
2	Wells [19], 2006	Spot 14	IHC score 2 vs. 0–1	5-year recurrence	14	53	0	21	100.00 (76.84–100.00)	28.38 (18.50–40.05)	0.21	1.00	1.40	0.00
3	Abduljabbar [12], 2015	PPARγ	IHC H-score ≥ 50 vs. < 50	Lymph node involvement	133	187	200	328	39.94 (34.64–45.42)	63.69 (59.37–67.85)	0.42	0.62	1.1	0.94
				HER2 positivity	29	279	97	415	23.02 (15.99–31.35)	59.80 (56.04–63.47)	0.09	0.81	0.57	1.29
				ER positivity	282	36	352	178	44.48 (40.57–48.44)	83.18 (77.48–87.93)	0.89	0.34	2.64	0.67
				PR positivity	217	94	259	252	45.59 (41.05–50.18)	72.83 (67.82–77.45)	0.70	0.49	1.68	0.75
				Ki-67 positivity	129	127	294	119	30.50 (26.14–35.13)	48.37 (41.98–54.81)	0.50	0.29	0.59	1.44
				15-year recurrence	115	203	224	303	33.92 (28.90–39.23)	59.88 (55.46–64.18)	0.36	0.58	0.85	1.10
4	Lucenay [16], 2016	PLIN2	IHC score 4–7 vs. 0–3	5-year recurrence	9	20	7	64	56.25 (29.88–80.25)	76.19 (65.65–84.81)	0.31	0.90	2.36	0.57
				10-year recurrence	11	18	7	64	61.11 (35.75–82.70)	78.05 (67.54–86.44)	0.38	0.90	2.78	0.50
				ER positivity	9	20	53	18	14.52 (6.86–25.78)	47.37 (30.98–64.18)	0.31	0.25	0.28	1.80
5	Feng [15], 2016	Elvol6	IHC score 2–3 vs. 0–1	Positive lymph node involvement	16	10	13	31	55.17 (35.69–73.55)	75.61 (59.70–87.64)	0.62	0.70	2.26	0.59
				ER positivity	17	9	23	21	42.50 (27.04–59.11)	70.00 (50.60–85.27)	0.65	0.48	1.42	0.82
				PR positivity	18	8	22	22	45.00 (29.26–61.51)	73.33 (54.11–87.72)	0.69	0.50	1.69	0.75
				HER2 positivity	10	16	18	26	35.71 (18.64–55.93)	61.90 (45.64–76.43)	0.38	0.59	0.94	1.04
				5-year recurrence	4	22	4	40	50.00 (15.70–84.30)	64.52 (51.34–76.26)	0.15	0.91	1.41	0.78
6	Cui [13], 2017	FASN	IHC score 5–12 vs. 0–4	Lymph node involvement	11	24	6	6	64.71 (38.33–85.79)	20.00 (7.71–38.57)	0.31	0.50	0.81	1.76
7	Choi [10], 2019	pS6K1	IHC score 1–3 vs. 0	5-year recurrence	67	177	21	163	76.14 (65.86–84.58)	47.94 (42.52–53.40)	0.27	0.89	1.46	0.50
				PR positivity	165	79	137	47	54.64 (48.83–60.35)	37.30 (28.85–46.36)	0.68	0.26	0.87	1.22
				HER2 positivity	43	201	21	163	67.19 (54.31–78.41)	44.78 (39.60–50.05)	0.18	0.89	1.22	0.73
8	Park [17], 2019	pS6K1	IHC score 1–3 vs. 0	Lymph node involvement	86	133	33	44	72.27 (63.32–80.08)	24.86 (18.68–31.90)	0.39	0.57	0.96	1.12
				5-year recurrence	19	200	1	76	95.00 (75.13–99.87)	27.54 (22.35–33.21)	0.09	0.99	1.31	0.18
				10-year recurrence	21	198	4	73	84.00 (63.92–95.46)	26.94 (21.75–32.64)	0.10	0.95	1.15	0.59
9	Dinarvand [14], 2019	Lipin-1	Tumor mRNA expression 2.28	ER positivity	21	5	19	7	52.50 (36.13–68.49)	58.33 (27.67–84.83)	0.81	0.27	1.26	0.81
				PR positivity	17	8	18	8	48.57 (31.38–66.01)	50.00 (24.65–75.35)	0.68	0.31	0.97	1.03
				HER positivity	6	10	10	26	37.50 (15.20–64.57)	72.22 (54.81–85.80)	0.38	0.72	1.35	0.87
				Ki-67 ≥ 20%	9	16	13	13	40.91 (20.71–63.65)	44.83 (26.45–64.31)	0.36	0.50	0.74	1.32
10	Song [18], 2020	Lipin-1	Median of immunoreactive score	5-year recurrence	11	18	2	23	84.62 (54.55–98.08)	56.10 (39.75–71.53)	0.38	0.92	1.93	0.27
				ER positivity	14	15	14	11	50.00 (30.65–69.35)	42.31 (23.35–63.08)	0.48	0.44	0.87	1.18
				PR positivity	14	15	8	17	63.64 (40.66–82.80)	53.12 (34.74–70.91)	0.48	0.68	1.36	0.68
				HER2 positivity	13	16	7	18	65.00 (40.78–84.61)	52.94 (35.13–70.22)	0.45	0.72	1.38	0.66
11	Yoshikawa [20], 2022	FASN	IHC score ≥ 120	Lymph node involvement	11	20	3	13	78.57 (49.20–95.34)	39.39 (22.91–57.86)	0.35	0.81	1.30	0.54
				Ki-67 positivity	19	18	18	3	51.35 (34.40–68.08)	14.29 (3.05–36.34)	0.51	0.14	0.60	3.41

*Abbreviations* FASN, fatty acid synthase; S14, Spot 14; pS6K1, phosphorylated ribosomal protein S6 kinase-1; PLIN2, adipophilin; Elovl6, elongation of long chain fatty acids family member 6; PPARγ, peroxisome proliferator-activated receptor-gamma; IHC, immunohistochemistry; ER, estrogen receptor; PR, progesterone receptor; HER2, human epidermal growth factor receptor 2; TP, true positive; FP, false positive; FN, false negative; TN, true negative; PPV, positive predictive value; NPV, negative predictive value; LR, likelihood ratio

### Meta-analysis

#### Association between adipogenesis and long-term outcomes

**Fig. 3:**
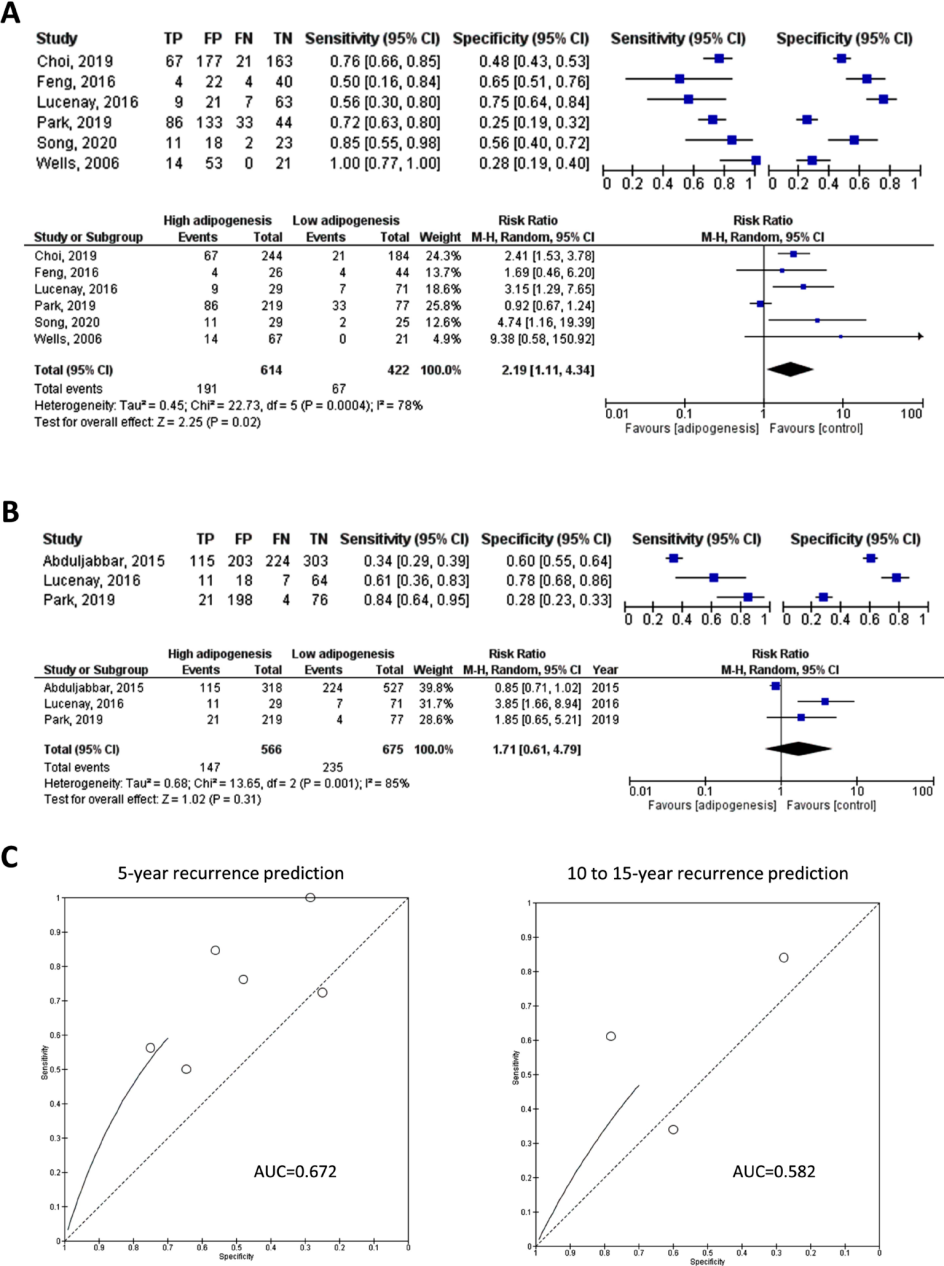
Diagnostic accuracy of tumor adipogenesis for long-term recurrence. (**A**) Diagnostic accuracy and meta-analysis for 5-year recurrence; (**B**) Diagnostic accuracy and meta-analysis for 10 to 15-year recurrence; c) Synthetic ROCs for meta-analysis for 5-year and 10-15-year recurrence prediction

A meta-analysis was conducted on 6 studies involving a total of 1,036 patients to assess the pooled predictive performance of adipogenesis biomarkers for 5-year breast cancer recurrence (Fig. 3). The sensitivity of these studies ranged from 0.50 to 1.00, while the specificity varied between 0.25 and 0.75, indicating significant heterogeneity in terms of cohort size and positive cases rate. The pooled diagnostic accuracy, as indicated by the risk ratio, was 2.19 (95% CI: 1.11–4.34) for patients with high adipogenesis biomarker expression compared to those with low adipogenesis status. The heterogeneity, as measured by I [2], was relatively large at 78%, which was also reflected in the synthetic ROC. Furthermore, the diagnostic accuracy of adipogenesis biomarkers for predicting 5-year recurrence was also validated by a summary ROC, indicating its significant predictive value with area under the curve (AUC) of 0.598 (Supplementary Fig. 1). In addition, three studies employed public databases consisting of 5,599 patients were run on meta-analysis for validating the effects of adipogenesis biomarkers in predicting 5-year cancer recurrence (Supplementary Tables 2–3). The overall effect was indicated by the odd ratio at 1.13 (95% CI: 1.01–1.27) (Supplementary Fig. 2A). Publication bias was relatively low to these include bioinformatics studies (Supplementary Fig. 2B).

Additionally, 3 studies with a total of 1,241 cases reported the predictive value of adipogenesis biomarkers for long-=term (10–15 years after treatment) cancer recurrence, and a meta-analysis was performed (Fig. 3). The heterogeneity in this analysis was even larger, with sensitivity ranging from 0.34 to 0.84 and specificity ranging 0.28 to 0.78, as illustrated by the SROC. The pooled diagnostic risk ratio of high adipogenesis status compared to low adipogenesis status was 1.71, but the overall effect was not statistically significant (95% CI: 0.61–4.79, *p* = 0.31).

#### Association between adipogenesis and cancer invasiveness

**Fig. 4:**
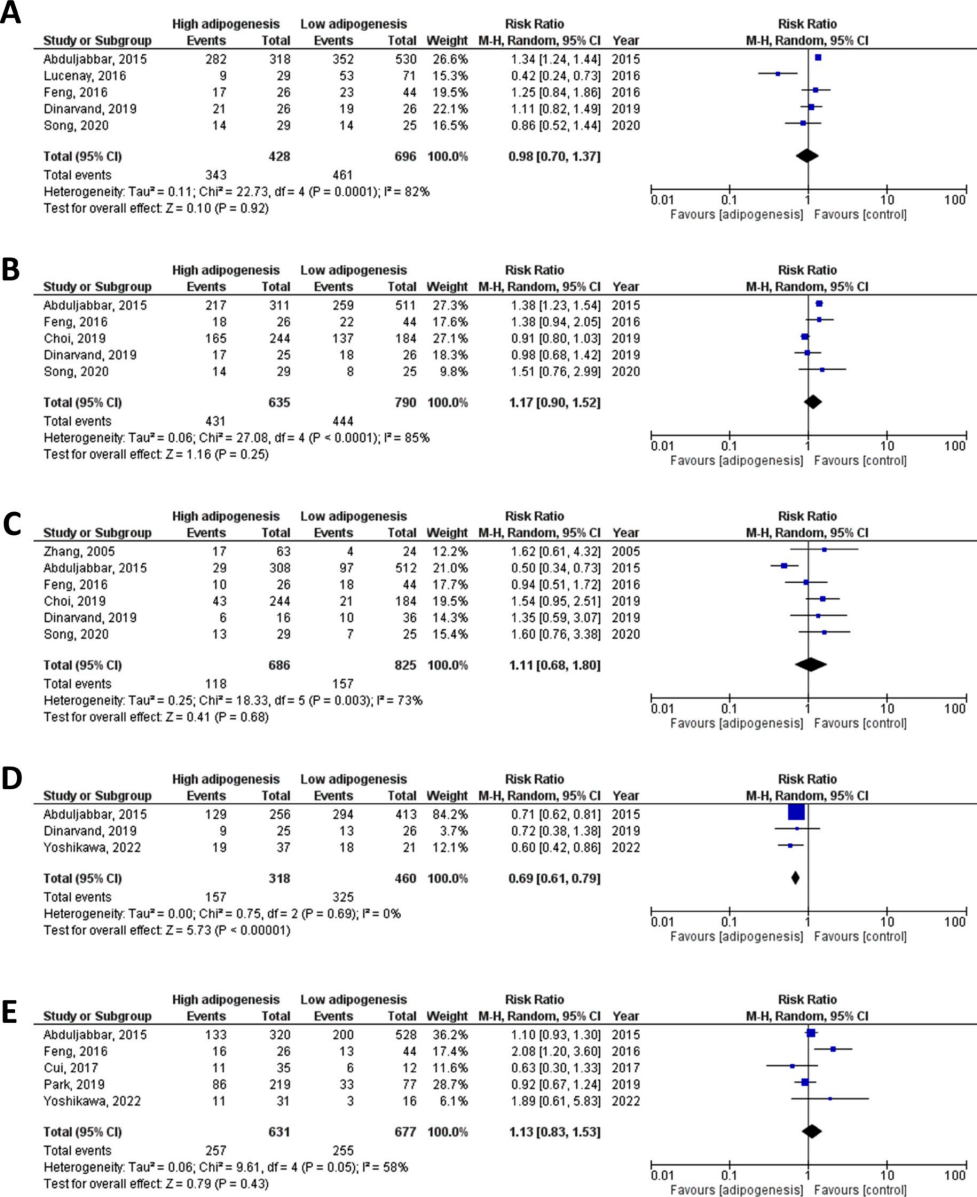
Subgroup analysis for correlation between tumor adipogenesis and empirical pathological markers. The pathological markers for correlation analysis include (**A**) ER; (**B**) PR; (**C**) HER2; (**D**) Ki-67. (**E**) Correlation with pathological finding of lymph node metastasis

The association between tumor adipogenesis biomarkers and indicators of cancer invasiveness, such as ki-67 positivity and the presence of lymph node metastasis, was analyzed using forest plots (Fig. 4). The results indicated a significant negative correlation between adipogenesis biomarker expression levels and ki-67, with a pooled risk ratio at 0.69 (95% CI: 0.61–0.79, *p* < 0.00001). The 3 studies included in this meta-analysis demonstrated high homogeneity with an I [2] of 0%. However, no correlation was found between adipogenesis biomarker expression and lymph node involvement status (RR = 1.13, *p* = 0.43).

#### Association between adipogenesis and empirical histological markers

A subgroup analysis was conducted to examine the potential co-effects of adipogenesis biomarkers and empirical outcome indicators for breast cancer, including ER, PR and HER2 positivity. The results showed no strong correlation between these variables (Fig. 4).

## Discussion

Our comprehensive study aimed to investigate the clinical significance of cancer cell adipogenesis in the diagnosis and prognosis of breast cancer after curative treatment. Histological examination of adipogenesis biomarkers in tumor tissues significantly predicted long-term overall and disease-free survival rates. Additionally, the cancer adipogenesis status was found to be independent of empirical markers such as ER, PR, and HER2. However, a negative correlation was observed between cancer adipogenesis status and cancer proliferation, as indicated by ki-67 expression. These findings suggest that cancer adipogenesis status, as determined by specific histological biomarkers, plays a crucial role in breast cancer prognosis and has the potential to enhance predictive models by incorporating it with traditional variables such as tumor biology and morphology. Future research should focus on conducting an in-depth analysis of cancer adipogenesis status, targeting a specific molecule to determine its predictive value in breast cancer outcomes. A prospective, large-scale, multi-center study should be conducted to establish consensus in the field.

Numerous studies have investigated the phenomenon of adipogenesis in cancer cells and its effects [30–33]. The impact of adipogenesis on the cancer cell biology indicates that increased adipogenesis promotes the proliferation, invasiveness, and metastasis of cancer cells [34]. Mechanistic research suggests that the increase in adipogenesis in tumor cells is mainly related to the abnormal regulation of key enzymes involved in lipid metabolism, increased expression of adipogenesis genes, disruption of signaling pathways responsible for carcinogenic transformation, and increased glycolysis related to tumorigenesis [32, 35]. Adipogenesis of cancer cells in breast cancer has also been observed. Previous studies have confirmed that most breast cancer cells exhibit the “adipogenic” phenotype, which is characterized by enhanced fatty acid synthesis activity for cell growth and survival [31]. Reprogramming of lipid metabolism is an important indicator of breast cancer [36]. Increasing large-scale clinical evidence-based research data has also confirmed that high adipogenesis levels in breast cancer are significantly associated with a high risk of breast cancer occurrence, recurrence, metastasis, drug resistance, and poor survival rate. However, due to the extremely complex lipid metabolism pathways in the tumorigenesis and progression of breast cancer, there are many biomarkers that can be used as adipogenesis indicators. Therefore, it is urgent to summarize and integrate the relationship between the broad concept of adipogenesis and breast cancer and to find evidence of using adipogenesis biomarkers to predict the outcomes. Our results indicate that several adipogenesis-related biomarkers are excellent predictors of breast cancer survival.

Adipogenic enzymes, particularly fatty acid synthase (FASN), play a crucial role in the regulation of metabolic pathways in breast cancer adipogenesis. Among the 11 studies included in our analysis, three investigated the role of FASN in breast cancer adipogenesis and its impact on prognosis. Overexpression of FASN, a key enzyme involved in *de novo* adipogenesis, was observed in breast cancer tissues and was associated with cancer progression, recurrence, poor prognosis, and pathological findings [22, 28, 29, 33]. Spot 14 which is required for FASN transcription, was reported associating with higher tumor grade, larger tumor size, and poor overall recurrence rates when its expression was upregulated [27]. Another important enzyme, lipin-1, acts as a phosphatidic acid phosphatase (PAP) and regulates the rate-limiting step in the triglyceride and phospholipid synthesis. Studies have reported that lipin-1 expression in breast cancer is correlated with pathological grade, tumor size, and p53 expression. Phosphorylated lipin-1, which enhances adipogenesis in breast cancer, is positively correlated with tumor size, lymph node metastasis, time to recurrence, and patient survival [14, 23]. Additionally, Elovl6, a long fatty acid elongase involved in *de novo* adipogenesis, was found to be upregulated and associated to lymph node involvement and short relapse-free survival in breast cancer [24]. The nuclear receptor superfamily member, peroxisome proliferator-activated receptor gamma (PPARγ), is also a promising prognostic marker associated with longer survival in breast cancer patients [12]. Furthermore, the expression of adipophilin (PLIN2), a specific marker for lipid droplet formation, was observed higher in HER2-positive and TNBC subtypes, but less in ER^+^PR^+^Ki67^low^ and ER^+^PR^+^Ki67^high^ subtypes, demonstrating its positive correlation with long-term cancer recurrence [25]. Lastly, phosphorylated ribosomal S6 kinase 1 (pS6K1), a downstream regulator of the mTOR pathway, was recently identified as a biomarker for adipogenesis, and its overexpression was associated with drug resistance and worse prognosis in breast cancer patients [20, 26].

While there is substantial evidence supporting the association between enhanced adipogenesis and poor outcomes in breast cancer, there is currently no consensus on a specified biomarker for clinical use. This lack of consensus can be attributed to the heterogeneity of breast cancer, including variations in pathology, genomic changes, and the tumor microenvironment (TME). These factors collectively impact the occurrence, progression, treatment response, and survival of breast cancer. Even patients with the same stage of pathological TNM may exhibit differences in treatment response and prognosis. Additionally, different breast cancer subtypes display significant variations in lipid metabolism. In this meta-analysis, the high heterogeneity among studies was observed and can be attributed to a variety of factors, including differences in sample sizes, the use of different molecules across studies, and variations in breast cancer subtypes and populations. Larger studies may overshadow the effects seen in smaller studies, potentially obscuring key findings if conflicting effects are present. The diverse selection of molecules used in these studies makes it difficult to establish a consistent cut-off point, and no single reliable biomarker has emerged as suitable for clinical application based on the pooled meta-analysis results. Moreover, the heterogeneity among these studies does not fully capture the role of adipogenesis within specific subgroups, such as different breast cancer subtypes and populations. Therefore, while a correlation between adipogenesis and breast cancer patients in general can be inferred, the findings may not be directly applicable to clinical practice at this time without further validation of biomarkers through large cohort studies.

Breast cancer subtypes demonstrate varying degrees of involvement in adipogenesis and lipid metabolism. Luminal subtypes predominantly rely on *de novo* adipogenesis, while the basal-like subtype utilizes exogenous fatty acids, synthesizes triacylglycerol and lipid droplets, and undergoes fatty acid oxidation [37]. In luminal breast cancer patients, PPARγ is an independent predictor of longer survival [12], while the overexpression of pS6K1 is associated with poor prognosis [20, 26]. Lipin1 has been identified as an independent prognostic factor for predicting worse prognosis, as its expression is independent of levels of ER and PR [14, 23]. In HER-2 positive patients, FASN expression is significantly higher than in other subtypes and is regulated by HER-2/neu signaling via the PI3K pathway [38]. Additionally, recent research has shown that HER2 directly phosphorylates and enhances FASN activity [39]. Adipogenesis is significantly lower in triple-negative breast cancer (TNBC), but high adipogenesis scores are significantly associated with worse survival in TNBC, but not in other subtypes [13]. Fatty acid metabolism and adipogenesis pathways are enriched in high-thermogenesis TNBC, which contributes to a tendency of worse survival [40]. Gene set enrichment analysis (GSEA) of protein genomic characteristics has shown a close correlation between baseline oxidative phosphorylation and fatty acid metabolism with chemotherapy resistance in TNBC, indicating that oxidative phosphorylation and fatty acid metabolism are potential driving factors [19].

We also explored the relationship between breast cancer ki-67 positivity and adipogenesis. One study demonstrated that high FASN expression was significantly correlated with a lower Ki-67 labeling index [28]. In another report, high FASN was significantly correlated with lymph node metastasis but not with pathological stage, ki-67 index, diseasefree survival, and overall survival in patients with TNBC [41]. There was a strong link between ki-67 and lipin-1, as lipin1 was negatively correlated with p53 mutation, while p53 mutant tumors exhibited higher expression of ki-67 compared to wild-type tumors. Similarly, PPARγ expression showed an inverse association with high proliferation status indicated by the ki-67 labeling index. Cox regression analysis revealed that PPARγ was an independent predictor of outcome [12]. Mechanistically, adipogenesis reflects the lipid metabolism activity and energy metabolism ability of cells, serving as the biomarker for energy source of tumor cell, but was not directly related to tumor behavior. Because of the Warburg effect, tumor cells prefer to utilize more rapid energy production pathway, rather than the more efficient process for their rapid proliferation. The role of adipogenesis in breast cancer cell Warburg effect is not fully understood, but the finding in this study suggests that breast cancer cell proliferation might be not dependent on cellular adipogenesis. However, the significant correlation of adipogenesis with long-term recurrence indicates that adipogenesis could be an independent biomarker for outcome prediction, in addition to current ER, PR, HER2, Ki-67. In-depth mechanism study focusing on the role of adipogenesis in breast cancer cell behavior should be performed to elucidate this phenomenon.

While adipogenesis has been shown to have significant clinical value in breast cancer, there is currently no single biomarker that can accurately represent adipogenesis. This may be due to the fact that tumor adipogenesis is regulated by different signaling pathways, influenced by various subtypes, clinical and pathological stages, populations, treatments, obesity, and sex hormone status. Furthermore, the identification of adipogenesis molecule expression cannot represent the real activity of the enzymes that involved in respective adipogenesis process, therefore hindering the direct correlation between cellular adipogenic activity and cell behavior. Hence, the direct correlation should be investigated in fine-tuned animal study. In addition, the methods of testing adipogenesis of breast cancer in these included studies were immunohistochemistry staining or real-time PCR of tumor tissue, which applicability in pre-surgery risk assessment was largely questioned. Therefore, correlating the expression in tissue with their status in liquid biopsy would be more promising in assisting decision-making before surgery, which would suggest neoadjuvant therapy for better outcome in high-risk patients. Moreover, the detailed regulatory mechanisms of adipogenesis in breast cancer invasion and metastasis are still not fully understood. Therefore, further in-depth basic research is needed Additionally, large-scale, multi-centric, randomized controlled clinical studies are particularly important for obtaining more reliable data on specific populations and cancer subtypes, which can aid in the development of new guidelines for more precise prediction models or biomarkers.

## Conclusion

In conclusion, adipogenesis in breast cancer has been shown to be a significant predict of long-term disease-free survival rate, independent of classic markers such as hormone receptors. Furthermore, adipogenesis biomarkers in breast cancer hold great potential improving current prediction models and serving as new diagnostic biomarkers and potential targets for breast cancer treatment.

## Electronic supplementary material

Below is the link to the electronic supplementary material.

Supplementary Material 1

## Data Availability

All data during this study are generated or analyzed from published articles and are included in this published article.
