# Supplementary material for: Adipogenesis biomarkers as the independent predictive factors for breast cancer recurrence: a systematic review and meta-analysis
Source: BMC Cancer. 2024 Sep 27;24:1181. doi: 10.1186/s12885-024-12931-1 (PMC11438415; doi:10.1186/s12885-024-12931-1)
Supplement: Supplementary file 1 — Supplementary Material 1 [file 12885_2024_12931_MOESM1_ESM.docx]

**Supplementary Figures**


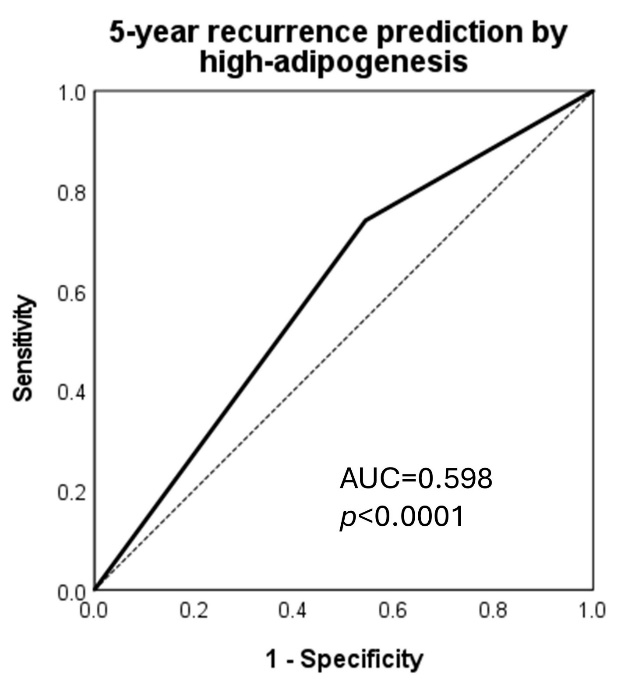


**Supplementary Figure 1.** ROC curve for adipogenesis-related molecules predicting 5-year recurrence.

AUC, area under the curve.


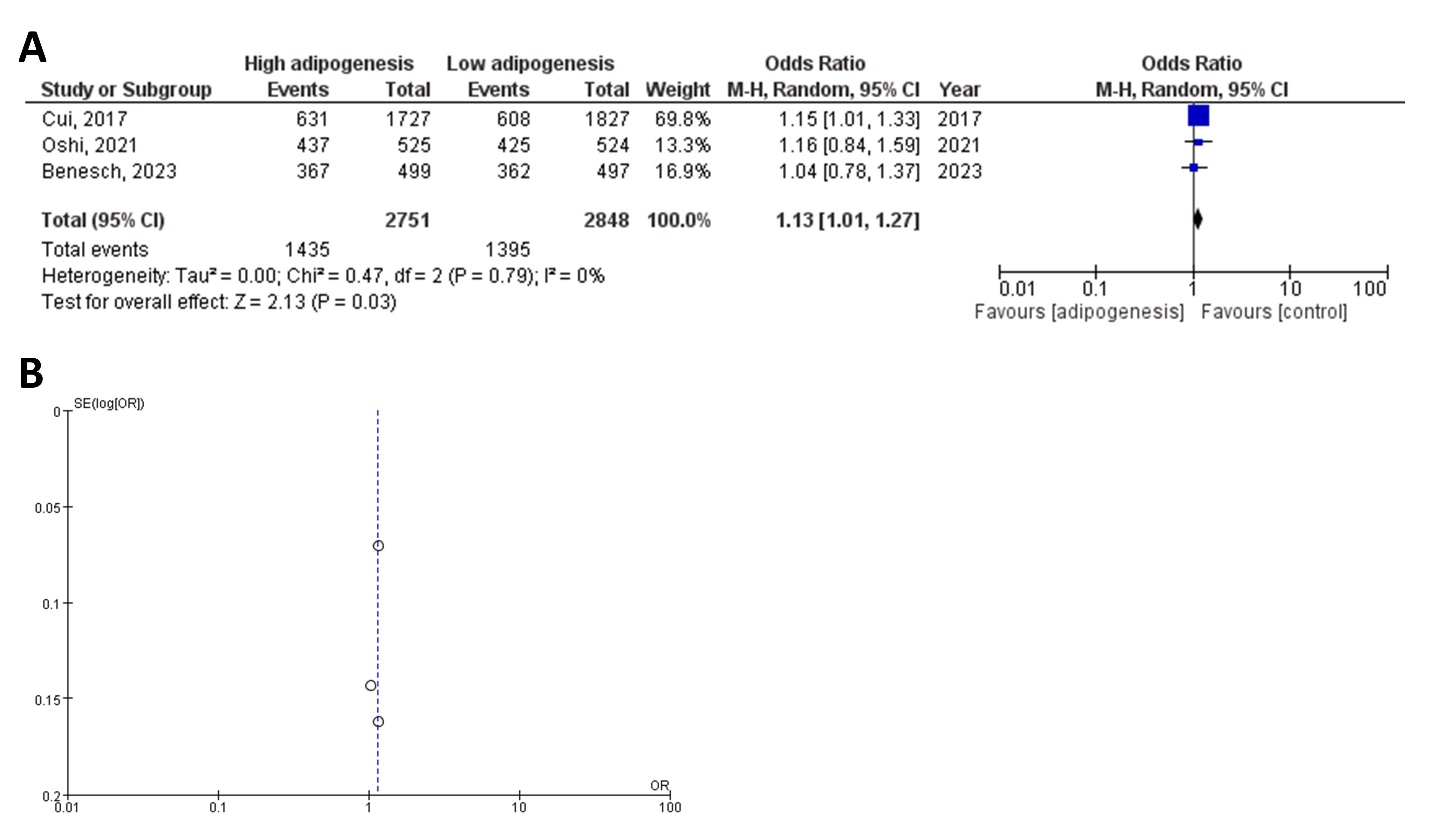


**Supplementary Figure 2**. Validation to diagnostic accuracy of tumor adipogenesis for long-term breast cancer recurrence from public database analysis.

A. Diagnostic accuracy and meta-analysis for 5-year recurrence by adipogenesis-related molecules from public database analysis; B. Funnel plots for publication bias analysis.

**Supplementary Tables**

**Supplementary Table 1**. Publication bias by Egger's test and Begg's test.

| Egger's test | | | | Begg's test | | | |
| --- | --- | --- | --- | --- | --- | --- | --- |
| beta1 | 1.839 | S.E. | 1.407 | Kendall’s score | 1 | S.E. | 5.323 |
| *t* | 1.306 | *df* | 4 | *z* | 0.188 | *p* | 0.851 |
| *p* | 0.261 |  |  |  |  |  |  |

**Supplementary Table 2**. Characteristics of included bioinformatics studies.

| **No.** | **Year** | **Author** | **Country** | **Data source** | **Biomarker** | **Total patients, n** | **Adipogenesis-high patients, n** | **Setting** | **Endpoints** |
| --- | --- | --- | --- | --- | --- | --- | --- | --- | --- |
| 1 | 2017 | Cui, et al. | China | Kmplot database | Tumor tissue FAS mRNA | 3554 | 1727 | All BC | 5-year recurrence |
| 2 | 2021 | Oshi, et al. | US, Japan | TCGA database | Tumor GSVA score of Hallmark Adipogenesis gene set | 1049 | 525 | All BC | 5-year recurrence |
| 3 | 2023 | Benesch, et al. | US, Japan | TCGA database | Tumor tissue PLPP2 | 996 | 499 | All BC | 5-year recurrence |

**Supplementary Table 3**. Statistics and diagnostic accuracy of included bioinformatics studies.

| **No.** | **Author, date** | **Biomarker** | **Cut-off** | **Outcome** | **TP** | **FP** | **FN** | **TN** | **Sensitivity (95% CI), %** | **Specificity (95% CI), %** | **PPV** | **NPV** | **+LR** | **-LR** |
| --- | --- | --- | --- | --- | --- | --- | --- | --- | --- | --- | --- | --- | --- | --- |
| 1 | Cui, 2017 | FAS | Median mRNA | 5-year recurrence | 631 | 1096 | 608 | 1219 | 50.93 (48.10 - 53.75) | 52.66 (50.60 - 54.71) | 0.37 | 0.67 | 1.08 | 0.93 |
| 2 | Oshi, 2021 | Tumor GSVA score | Median mRNA | 5-year recurrence | 437 | 88 | 425 | 99 | 50.70 (47.30 - 54.08) | 52.94 (45.52 -60.26) | 0.83 | 0.19 | 1.08 | 0.93 |
| 3 | Benesch, 2023 | PLPP2 | Median mRNA | 5-year recurrence | 367 | 132 | 362 | 135 | 50.34 (46.65 - 54.03) | 50.56 (44.40 - 56.71) | 0.74 | 0.27 | 1.02 | 0.98 |
